# Tides in Inland Waters

**Published:** 1883-05

**Authors:** 


Tides in Inland Waters.—It is found by the survey of the Great Lakes that there is a slight tide in them, but not of sufficient extent to be noticeable without special care; the amount of rise and fall not exceeding two inches.

